# Mating and aggressive behaviour of *Brachyrhaphis olomina* (Cyprinodontiformes: Poeciliidae)

**DOI:** 10.1007/s10164-017-0523-y

**Published:** 2017-08-21

**Authors:** Carlos A. Garita-Alvarado, Beatriz Naranjo-Elizondo, Gilbert Barrantes

**Affiliations:** 1 Sistema de Estudios de Posgrado en Biología, Universidad de Costa Rica, San José, 11501-2060 Costa Rica; 2 Escuela de Biología, Universidad de Costa Rica, San José, 11501-2060 Costa Rica; 3 Centro de Investigación en Ciencias del Mar y Limnología, Universidad de Costa Rica, San José, 11501-2060 Costa Rica; 4 Instituto de Biología, Universidad Nacional Autónoma de México, Tercer Circuito Exterior S/N., C.P. 04510 CDMX, México

**Keywords:** Behaviour description, *Brachyrhaphis*, Courtship, Colour changes, Mature ovum, Pregnant female

## Abstract

Despite the increasing interest in the use of intromittent male genitalia and coercive mating behaviour in poeciliids, detailed studies of the mating behaviour of most species in this family are lacking. We describe here the mating and aggressive behaviours of *Brachyrhaphis olomina*, and correlate them with the condition of the female’s ovum and embryos (immature, mature and pregnant). *B. olomina* performed a wide range of aggressive (sidle spread, tail beating, coordinate) and mating behaviours (approximation, touch, lateral display, touch-lateral display). Some behaviours (e.g. tail beating) are shared with other poeciliids, but two sexual behaviours (touch and lateral display) and one aggressive (coordinate) behaviour may be unique to *B. olomina* and were not reported in a previous study. Differences in male behaviour when paired with a female with mature ovum (more mating displays, no agonistic movements) suggest that males detect the female’s reproductive condition from some distance. The distinctive nature of mating behaviour in *B. olomina* highlights the importance of studying different species to have a better understanding of the evolution of mating and aggressive behaviours in poeciliids. Digital video images related to the article are available at http://www.momo-p.com/showdetail-e.php?movieid=momo170720bo01a, http://www.momo-p.com/showdetail-e.php?movieid=momo170720bo02a and http://www.momo-p.com/showdetail-e.php?movieid=momo170720bo03a.

## Introduction

The live-bearing fish subfamily Poeciliinae (Ghedotti 2000) is a diverse group of small fishes, in which males are unusual in having a gonopodium (modification of the anal fin rays 3, 4 and 5) used to transfer sperm to females during copulation. Poeciliid mating behaviour has attracted the interest of biologists for decades (e.g. Schlosberg et al. 1949; Rosen and Tucker 1961; Kolluru et al. 2014). Traditionally, two male mating tactics have been described: courtship, in which males perform stereotyped behaviours to persuade females to copulate; and coercive mating (sneak copulation), a fertilization strategy that bypasses the female’s consent (Bisazza 1993; Martin et al. 2010). Some studies have shown that the coercive male mating tactic is associated with a longer gonopodium in some genera, while short gonopodium species use either courtship or both tactics (Rosen and Tucker 1961; Langerhans 2011). For instance, a positive correlation between coercive mating tactic and gonopodium length was found in *Poecilia reticulata* (Reynolds et al. 1993) and Bisazza et al. (1997) studied the evolution of both male mating strategies in eight poeciliid genera finding that sneak copulation was the primitive mating strategy of the family [but see Lucinda and Reis (2005), and Pollux et al. (2014) for other hypothesis about the relationships among poeciliid genera and tribes].

Despite this interest in elucidating the evolution of sexual strategies and sexual conflict of male mating tactics (e.g. Plath et al. 2007; Wang et al. 2015), studies that detail specific movements and behaviours during courtship and mating are still lacking for most species in this group (Kolluru et al. 2014). Most specific studies have focussed on a few species in the genera *Poecilia, Xiphohorus* and *Gambusia*; within a family with approximately 28 genera (Langerhans 2011), limiting the scope of comparative studies of the mating behaviour patterns in the family.

The specific mating behaviour of the few poeciliids studied is quite variable; the behaviours displayed during precopulation usually involve specific movements of the male’s body and fins, while females may or may not apparently present specific responses (Schlosberg et al. 1949; Clark et al. 1954; Baerends et al. 1955; Liley 1966; Parzefall 1969; Peden 1972; Mojica 1996; Kolluru et al.^2014^). For example, Schlosberg et al. (1949) and Clark et al. (1954) described the mating behaviour and its differences between *Xiphophorus helleri* and *Xiphophorus maculatus*. Both species show ‘arcing’ (Schlosberg et al. 1949) or ‘backing’ (Clark et al. 1954) behaviour, in which the male stays in front of the female with its fins erect and quivering, with the body often rigidly bent in a sigmoid pattern, and then swims backward toward the female. This behaviour is more exaggerated in *X. helleri* due to the long sword-like extension of the tail and because the male often starts the display at a long distance from the female (Clark et al. 1954).

Liley (1966) described the mating behaviour of males and females of four sympatric species of *Poecilia*; each showed ‘elaborate patterns of courtship movements which are strikingly distinct and species-specific’. *P. reticulata* showed the ‘sigmoid display’ in which the male stays in front of the female, quivering its body in a sigmoid position, with dorsal and caudal fins either folded or extended; while *Poecilia vivipara* only showed the ‘nudge’ behaviour in which the male’s snout contacts and vibrates gently against the female’s cloaca (Liley 1966). The other two species, *Poecilia picta* and *Poecilia parae*, showed more complex courtship behaviours in which the male usually moves vigorously around the female (‘orbit’-‘dance’ and ‘dart’-‘gyrate’, respectively for each species) (Liley 1966). Parzefall (1969) also described and compared the courtship and aggressive behaviour of *Poecilia latipinna, Poecilia velifera* and *Poecilia mexicana*. Additionally, Peden (1972, 1975) studied the function of the gonopodial parts and behavioural patterns during copulation in several species of *Gambusia*, showing that the direction of the gonopodial thrust of males during copulation correlates with the shape of the female genitalia. Recently, Kolluru et al. (2014) described the courtship of *Girardinus metallicus*, a species for which the courtship display was previously thought to be absent. During the display in this species, the male raises its head and swims around the female’s field of vision showing its black ventral surface and gonopodium extended at an angle of 90° relative to its body (Kolluru et al. 2014).

The mating behaviour has also been studied with some detail in five species of *Brachyrhaphis* (Mojica 1996). With the exception of *Brachyrhaphis hartwegi*, males of all species showed specific mating behaviours; for instance, *Brachyrhaphis roseni* showed three unique behaviours (‘tandem’, ‘flank’ and ‘swim-back’). The male *Brachyrhaphis rhabdophora* swims rapidly toward the female’s vent turning rapidly from the female, forming a figure-of-eight (Mojica 1996). This behaviour was not seen in other species of *Brachyrhaphis*, but according to Mojica (1996) it resembles the Dance behaviour of *Poecilia picta* (Liley 1966). However, Mojica (1996) did not mention the origin of the population of *Brachyrhaphis rhabdophora* studied, and since the author considered *Brachyrhaphis rhabdophora* and *Brachyrhaphis olomina* as the same species, it is not clear which is the mating behaviour of each one. However, as noted by Mojica (1996), the observations were not systematic and were conducted in stock tanks, which makes quantitative analysis difficult.

Some studies on mating behaviour in poeciliids have reported that males can detect female reproductive condition by non-visual cues (e.g. pheromones), and they show preference or increase their sexual activity towards fertile females in *Poecilia* (Parzefall 1973; Crow and Liley 1979; Brett and Grosse 1982; Farr and Travis 1986) and *Gambusia* (Park and Propper 2002). Hence, considering that sexual selection predicts that both sexes are selected to maximize their reproductive success (Trivers 1972), a reasonable expectation for males would be that they select those females that render a higher reproductive success over those females whose eggs are unlikely to be fertilized (Park and Propper 2002). For females, attracting males might increase inter-male competition and indirectly favour female’s reproductive success (Crow and Liley 1979). However, as noted by Park and Propper (2002), a full understanding of the reproductive success of poeciliids is incomplete if information on females advertising their fertility is unknown.

Regarding the few detailed studies on mating behaviour in poeciliids, the uncertainty on the mating behaviour of *B. olomina* and the lack of information of the effect of female reproductive condition on male behaviour for most poeciliid genera (including *Brachyrhaphis*), in this study we have three objectives: to describe in detail and quantify the mating and aggressive behaviour of males and females of *B. olomina*, to compare the behaviours of *B. olomina* with behaviours described for other poeciliids, and to answer whether there is any effect of the reproductive condition of females on male mating behaviour in this species.

## Materials and methods

### Study species and fish maintenance

*Brachyrhaphis olomina* inhabits the north Pacific and some drainage basins of the Atlantic versant of Costa Rica (Bussing 1998; Angulo et al. 2013). Some recent studies have considered the populations of north-western Pacific of Costa Rica to in fact be those of *B. rhabdophora* (e.g. Johnson 2002; Langerhans and DeWitt 2004; Hassell et al. 2012; Ingley et al. 2015; Ingley and Johnson 2016), a species inhabiting the central and south Pacific of Costa Rica, which is morphologically (Bussing 1998; Angulo et al. 2013), genetically (Mojica 1996) and behaviourally (Garita-Alvarado unpublished data) distinct from *B. olomina.* We captured *B. olomina* using seine and hand nets in the Río Cañas (10°25′36.58″N, 85° 0′3.54″W) in the lowlands of the Pacific versant in north-western Costa Rica, on 28 February 2013. Several predatory fish are found in this locality, such as ‘guapotes’ (*Parachromis dovii*) and guavinas’ (*Gobiomorus maculatus*), known to prey on poeciliids (Bussing 1998). Sexual behaviour is affected by predation pressure (Farr 1975; Garita-Alvarado, unpublished data) in poeciliids. We captured specimens under permits of the Sistema Nacional de Áreas de Conservación (SINAC) and the Ministerio de Ambiente y Energía of Costa Rica (permits 007-2013-SINAC and SINAC-GASP-PI-R-072-2014).

We kept *B. olomina* at the Centro de Investigación en Ciencias del Mar y Limnología of the Universidad de Costa Rica under a natural light:dark cycle, and fed them once a day with commercial flakes. We kept male and female fish in an aquarium 40 cm × 50 cm × 100 cm long for a 1-week acclimation period. We then separated males by placing them into another aquarium 30 cm × 35 cm × 75 cm long for a second acclimation period of 1 week before beginning the behavioural trials. Because of the high tendency of poeciliids to copulate (Plath et al. 2007), we assumed that females were not virgins, which allowed us to included females of different reproductive state in the analysis (see the next section for an explanation).

### Behavioural trials and data analysis

**Fig. 1a–d:**
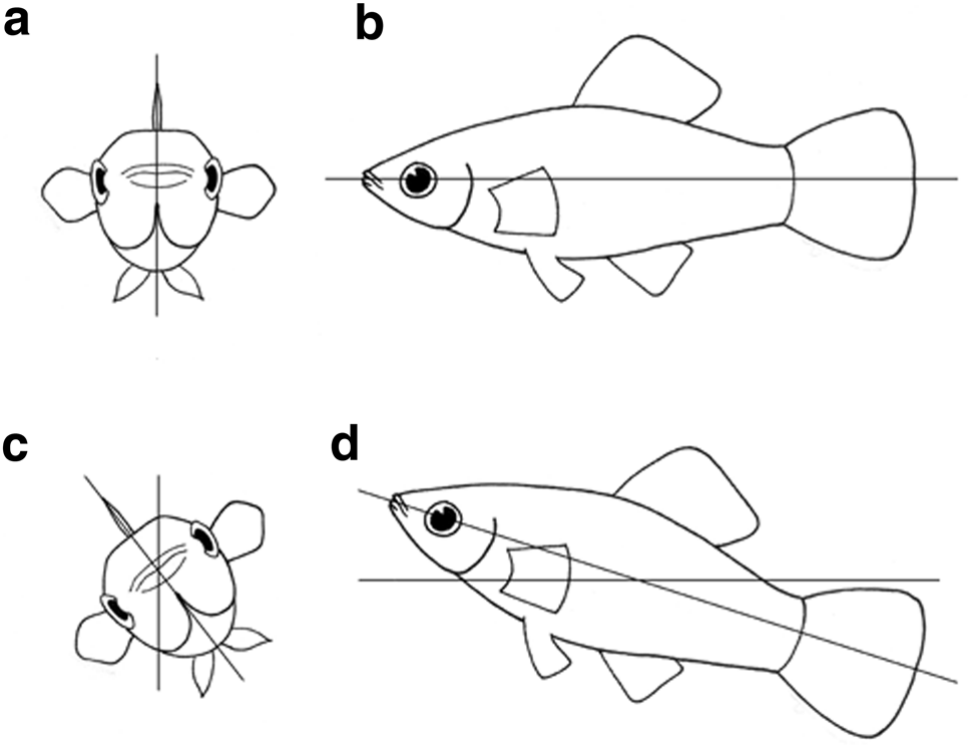
Fish longitudinal axis and movements of fish used in this study. The fish in **a** is perpendicular to the longitudinal axis in **b**. The fish in **c** rolls with respect to the longitudinal axis in **a**. The longitudinal axis of the fish in **d** is inclined with the head upward with respect to the longitudinal axis of the fish in **b**

We randomly paired 20 males with 20 females; females were longer than males in all cases. Each testing pair was isolated in an aquarium 25 cm × 20 cm × 40 cm long with a submersible filtration pump at one side; the males and females were separated by an opaque plastic partition for 1 day (we randomized the filtration pump between the male and female side). The next day we removed the partition and recorded the pair’s behaviours with a digital video camera (Sony HDR-SR11; 29 frames per second) for 20 min, beginning when the pair made visual contact. We referred to the longitudinal axes of the fish (Fig. 1) to describe their movements and behaviour. From the videos, we defined each behaviour, counted its frequency and established the sequence of behaviours (see “Results” section for behavioural descriptions). When possible we followed the behavioural classifications of Mojica (1996).

After each trail, we humanely euthanised each pair of fish by putting them into iced water (Leary et al. 2013). We preserved the fish by placing them first in a 10% formalin solution for at least 7 days and then transferred them to 70% ethanol. We measured the standard length of each fish (male mean 2.43 ± 0.43 cm, range 1.76–3.15 cm; female mean 3.01 ± 0.47 cm, range 2.23–4.09 cm) and dissected the females to determine their reproductive condition based on ovum and embryos categories following Haynes (1995): immature ovum (stages 1–2, *n* = 9); mature ovum (stage 3, *n* = 4); and pregnant (stages 4 to 11, *n* = 7). We tested the effect of the female reproductive condition and size of both males and females on the male behavioural response variables, and used the pair identity as a random factor, using a general linear mixed model (GLMM) with a negative binomial distribution (R statistical language, packages nlme and MAS). For each response variable, we ran two models; in the first, we included the interaction of male × female size, but if the interaction was not significant, we ran a second model without this interaction. We included in the analyses the following behaviours as response variables (see “Results” section for the detailed descriptions of behaviours): (1) approximation + approximation attempt, and (2) touch + touch attempt + lateral display + touch-lateral display (T–LD) (all variables correspond to male behaviours obtained from the 20 min each pair was recorded). Additionally, we conducted paired *t*-tests between male and females of each pair to test for differences in sidle spread and tail beating (response variables). All analyses were conducted in the R Statistical Language (version 3.03: http://cran.r-project.org). Additionally to the trials, we video-recorded a casual observation of an aggressive interaction between two females (when they were isolated from males prior to the trails).

## Results

### Description of aggressive behaviours performed in both sexes

#### Sidle spread

**Fig. 2:**
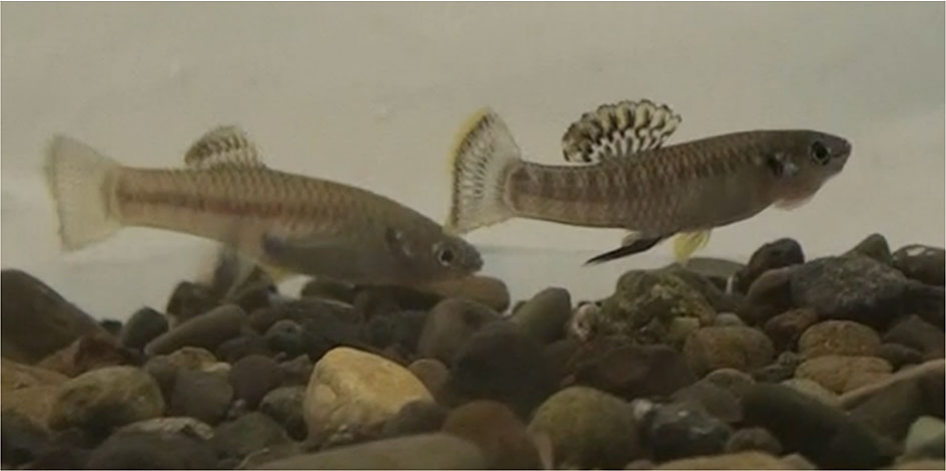
Aggressive behaviours of *Brachyrhaphis olomina* described in this study; frame showing sidle spread by a male (http://www.momo-p.com/showdetail-e.php?movieid=momo170720bo01a)

**Fig. 3:**
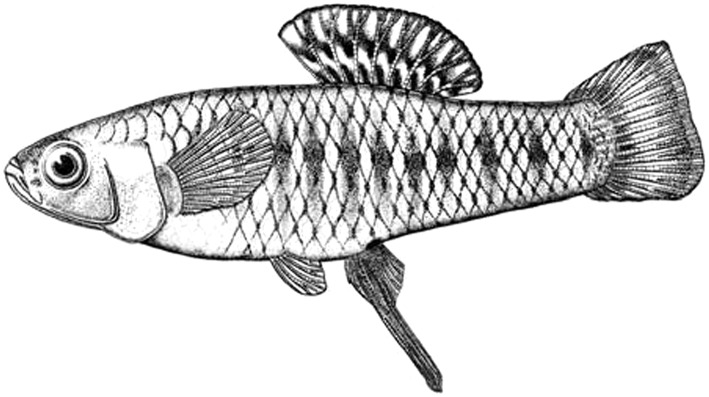
Male *Brachyrhaphis olomina* performing the spread position. See the description of sidle spread behaviour for details

In sidle spread (Fig. 2; counter no. 00:01–00:30 in the video image: http://www.momo-p.com/showdetail-e.php?movieid=momo170720bo01a), the signaller performs a fast swimming movement of about one to three body lengths (BL) to a position in front of the receiver fish; during this movement, the male’s gonopodium or the anal fin of the female is folded close to the body and the dorsal fin is either folded or partially extended. When this movement ends, the two fish are separated by between one or two BLs from each other and their longitudinal axes are at an angle that varies from perpendicular to about 45°. The signaller also maintains a weak sigmoid position (Liley 1966), the caudal fin is fluttering, the pectoral fins move vigorously, the dorsal and pelvic fins are fully extended, the brachiostegal rays are sometimes expanded and sometimes the signaller rolls slightly on its longitudinal axis so that its ventral surface is closer to the receiver. The signalling fish usually remains in this position for only a second or less (depending on the activity of the receiver) with the anal fin completely extended in signalling females and in signalling males the gonopodium is maintained forward, making an angle of between 90° and 45° with respect to the longitudinal axis of the signaller (in other cases the anal fin was close to the body). The ‘behaviour spread’ (Fig. 3) described by Mojica (1996) for *Brachyrhaphis* spp. has some similarities to the sidle spread, but differs in several details: it lacks the initial fast movement, the position of the signaller fish is not at a consistent angle to the receiver and there is no rolling along the longitudinal axis. We did not include the spread position as a distinct behaviour in our analyses because this behaviour sometimes forms part of other behaviours and is not a separate behaviour in itself (see below).

#### Tail beating

Tail beating is shown in Fig. 2 (counter no. 00:30–00:56 in the video image: http://www.momo-p.com/showdetail-e.php?movieid=momo170720bo01a). The signalling fish is in the spread position at a distance shorter than three BL, and lashes its tail, moving its entire body with the dorsal fin completely extended, the opercula sometimes fluttering and the brachiostegal rays of the signaller sometimes expanded. In signalling females, the anal fin is completely extended, and in signalling males the gonopodium is maintained forward at an angle between 90° and 45° with respect to its longitudinal axis, or is sometimes close to the body. The signaller performs one to six lashes in a quick sequence (henceforth ‘tail beating series’). When both fish are very close to each other, the tail contacts the flanks (Liley 1966) or the head of the receiver.

#### Coordinate

In the coordinate behaviour (Fig. 2; counter no. 00:56–01:08 in the video image: http://www.momo-p.com/showdetail-e.php?movieid=momo170720bo01a), the pair remain parallel to each other nearly motionless in the spread position, between one and two BL apart. They then move quickly forward simultaneously at about two to six BL apart. While moving forward, both fish maintain their dorsal and caudal fins completely extended, their bodies show a weak sigmoid position, the brachiostegal rays are extended; in the female the anal fin is fully extended, while the male directs the gonopodium forward at an angle between 90° and 45° with respect to the longitudinal axis. Both fish stop at the same time, maintaining the positions of their fins for a short period of time (usually less than 1 s) until they perform other behaviours.

#### Agonistic movement

**Fig. 4:**
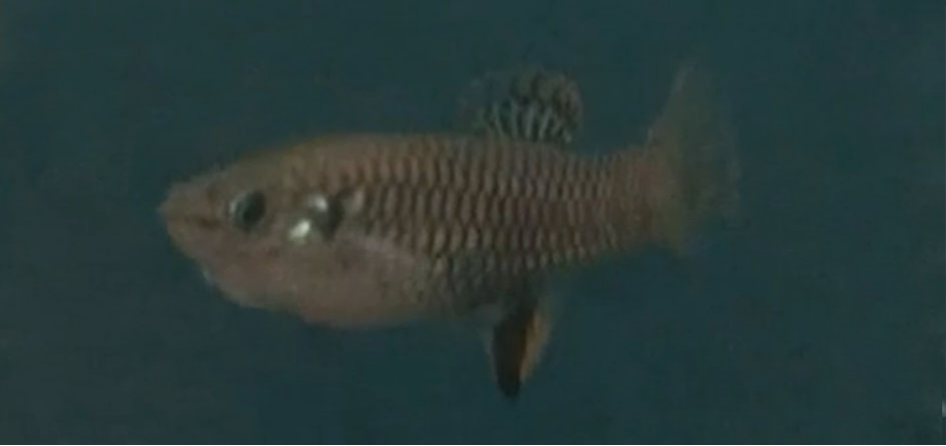
Female–female aggressive behaviour of *B. olomina*: frame showing the darkened and expanded anal blotch (http://www.momo-p.com/showdetail-e.php?movieid=momo170720bo02a)

In the agonistic movement (Fig. 2; counter no. 01:08–01:22 in the video image: http://www.momo-p.com/showdetail-e.php?movieid=momo170720bo01a, Fig. 4; http://www.momo-p.com/showdetail-e.php?movieid=momo170720bo02a), the male or female swims quickly, directly toward the receiver; sometimes the aggressor bites or hits the other individual, but usually the receiver evades the contact as it apparently perceives the approaching aggressor.

### Description of mating behaviours

#### Approximation and approximation attempt

**Fig. 5:**
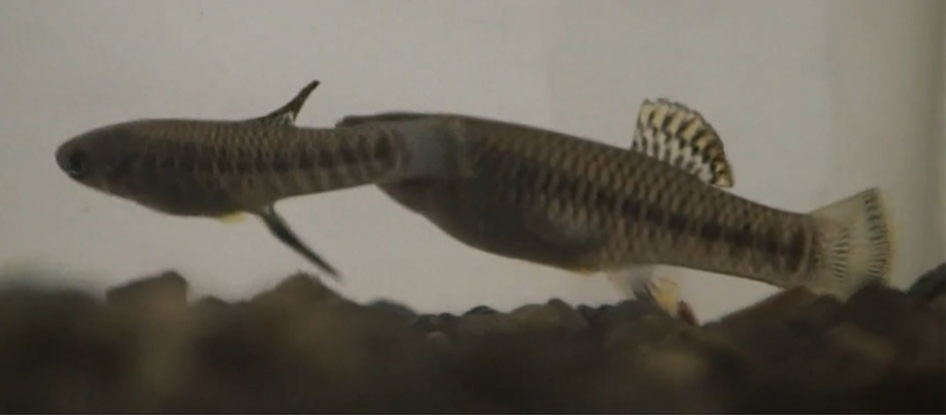
Mating behaviour of *B. olomina*: frame showing lateral display of a male (http://www.momo-p.com/showdetail-e.php?movieid=momo170720bo03a)

In the approximation and approximation attempt (Fig. 5; counter no. 00:01–00:29 in the video image: http://www.momo-p.com/showdetail-e.php?movieid=momo170720bo03a), the male swims toward the female from a distance of one to five BL, touching the ventral side of female with the mouth, the dorsal part of the head and/or the dorsal fin. When the male is below the female, the female usually either turns rapidly to face the male, or swims away. Males approach the female from many different directions, and contact the female at nearly any possible angle with respect to the female’s longitudinal axis. The contact with the female is brief, since the male does not pause while swimming below it. After contacting the female, the male faces the female again and usually performs approximation again (often several times). An approximation attempt occurs when the male does not come in contact with the female, either because the female moves or because the male does not get close enough.

#### Touch and touch attempt

In the touch and touch attempt (Fig. 5; counter no. 00:30–00:50 in the video image: http://www.momo-p.com/showdetail-e.php?movieid=momo170720bo03a), the male faces the female from different angles with respect to the longitudinal axis of the female and is above or below the female, at two to five BL away. When the female is above or below the male, the male inclines its longitudinal axis to face the female more directly (inclining the head up when the female is above or down when it is below). The male swims quickly to a position below the female, parallel to the longitudinal axis of the female (both facing the same direction), and when the male is almost below the female, the male extends its dorsal fin and then touches the belly and ventral side of the female head with the upper part of its head while moving forward. The male head is inclined upward at an angle of about 45° along the longitudinal axis while touching the female, and sometimes the male dorsal fin touches the female anal fin and belly. The male continues moving until it is in front of the female, stays some instants there and then may perform other behaviours. A touch attempt occurs when the male fails to make contact with the female (because the female moves or because the male does not get close enough).

#### Lateral display

In the lateral display (Fig. 5; counter no. 00:51–01:53 in the video image: http://www.momo-p.com/showdetail-e.php?movieid=momo170720bo03a), the male displays its side in front of the female at an angle varying from almost perpendicular to about 45° with respect to the female’s longitudinal axis, and at about one BL in front of the female with the body rolled to about 45° on its longitudinal axis. The dorsal fin of the male is either folded or unfolded, the caudal fin partially folded, the pelvic fins close to the body and the gonopodium extended toward the female at an angle of about 90° with respect to its longitudinal axis. The male moves slowly toward the female while moving its caudal fin vigorously; then the male quickly changes its position showing the other side of the body and moving toward the female again. This change of position, from one side to the other, occurs up to 11 times. If the female moves forward or backward while the male is displaying, the male follows the female, always showing one of its sides. Finally, the male often swims quickly to the ventral side of the female and occasionally touches her. The touch behaviour is often followed by the lateral display (henceforth T-LD) (Fig. 5; counter no. 01:53–02:47 in the video image: http://www.momo-p.com/showdetail-e.php?movieid=momo170720bo03a).

#### Gonopodial swing

In the gonopodial swing (Fig. 5; counter no. 02:48–03:02 in the video image: http://www.momo-p.com/showdetail-e.php?movieid=momo170720bo03a), the male moves the gonopodium laterally and then forward at nearly 180° with respect to the longitudinal axis. As the gonopodium is moving at an angle of about 90° with respect to the longitudinal axis, the pelvic fin (of the side where the gonopodium moved laterally) moves backward and then forward to hold the gonopodium in position as it swings forward. After this movement of the gonopodium, the male occasionally arches or quickly jerks its body. The male extends totally or partially the dorsal fin while moving the gonopodium.

#### Copulation and copulation attempt

In copulation and during a copulation attempt (Fig. 5; counter no. 03:03–03:09 in the video image: http://www.momo-p.com/showdetail-e.php?movieid=momo170720bo03a), the male swims quickly toward the ventral part of the female while moving the gonopodium forward (as in the “Gonopodial swing”). When the male is almost below the female, it rolls its body on the longitudinal axis, inclining the head upward at nearly 90° to reach the female’s genital opening with the gonopodium. As the male continues moving upward, the tip of the gonopodium makes brief contact with the female’s genital opening. A copulation attempt occurs when the gonopodium does not contact the female, because the female moves or because the male does not get close enough.

### Sequence of behaviours, frequency and colour changes

The frequency, range, and percentage of pairs that performed each of the behaviours described, as well as those behaviours related to the reproductive condition of the female, are included in Table 1. In general, we categorized behaviours according to either an aggressive or a mating context based on similarities with previously described behaviours for poeciliids in other studies (see the “Discussion” section for a comparison between behaviours). After the plastic partition of the tank was removed and the pair made visual contact, the male usually approached the female; in 65% of the cases the pair performed aggressive behaviour (sidle spread and tail beating), while in 35% the male performed some type of sexual behaviour (approximation and approximation attempt—‘approximation phase’). Males performed more aggressive behaviours than females (sidle spread, *t* = 3.21, *df* = 19, *p* = 0.004; tail beating, *t* = 2.12, *df* = 19, *p* = 0.04; SS + TB males in Table 1), and usually performed them before the female; females often responded with sidle spread, tail beating or agonistic movement. Sometimes during the initial interactions, the pair also performed the coordinate behaviour. Approximation and approximation attempt were also performed after or during aggressive displays and were the most common mating behaviours of males (up to 23 per minute performed by a male on one occasion; Table 1). The female often performed aggressive behaviours while the male was performing approximation.

**Table 1:** Mean and range of aggressive and mating behaviours performed by females and/or males during trials in which the reproductive condition of females varied from immature to mature or pregnant

Behaviour	Immature ovum (*n* = 9)			Mature ovum (*n* = 4)			Pregnant (*n* = 7)			All stages (*n* = 20)	
	Mean	Range	% Shown	Mean	Range	% Shown	Mean	Range	% Shown	Mean	Range
Aggressive behaviour											
Sidle spread (SS) ♀	2.11	0–10	44.4	0	0	0	0	0	0	0.95	0–10
Tail beating series (TB) ♀	3.89	0–19	22.2	1	0–4	25	0	0–1	0	2	0–19
SS + TB ♀	6.00	0–26	44.4	1	0–4	25	0.14	0–1	14.3	2.95	0–26
Sidle spread ♂	18.11	0–63	77.8	7.5	0–17	75	17.57	0–63	57.1	15.8	0–63
Tail beating series ♂	10.44	0–38	77.8	0.5	0–1	50	11.86	0–53	57.1	8.95	0–53
SS + TB ♂	28.56	0–98	77.8	8	0–18	75	29.43	0–75	57.1	24.75	0–98
Coordinate	1.33	0–8	33.3	0	0	0	0.43	0–2	28.6	0.75	0–8
Agonistic movement ♂	12.78	0–113	22.2	0	0	0	9.14	0–64	14.3	8.95	0–113
Agonistic movement ♀	67.89	0–156	88.9	61	53–95	100	38.14	0–114	57.1	56.1	0–156
Mating behaviour											
Approximation attempt (AA)	14.44	0–46	77.8	25.25	5–53	100	12.43	0–36	71.4	15.9	0–53
Approximation (A)	25.78	0–86	88.9	76.25	22–144	100	17.29	0–35	71.4	32.9	0–144
AA + A	40.22	0–132	88.9	101.5	48–197	100	29.71	0–71	71.4	48.8	0–197
Touch attempt (TA)	0.11	0–1	11.1	0.75	0–2	50	1.29	0–7	42.9	0.65	0–7
Touch (T)	1.78	0–9	44.4	7.25	0–21	75	2	0–8	71.4	2.95	0–21
Lateral display (LD)	0.33	0–2	22.2	1	0–2	50	0.43	0–2	28.6	0.5	0–2
Touch-lateral display (T-LD)	3.78	0–11	77.8	6	3–9	100	1.43	0–3	71.4	3.4	0–11
TA + T+LD + T-LD	6.00	0–16	77.8	15	8–28	100	5.14	0–16	85.7	7.5	0–28
Gonopodial swing	2.56	0–6	66.7	3.25	0–11	75	4.29	0–11	57.1	3.3	0–11
Copulation attempt	0.22	0–1	22.2	0.25	0–1	25	0.14	0–1	14.3	0.15	0–1

Percentage of trails where behaviours were performed (*% Shown*) are given according to the reproductive condition of females

**Fig. 6a, b:**
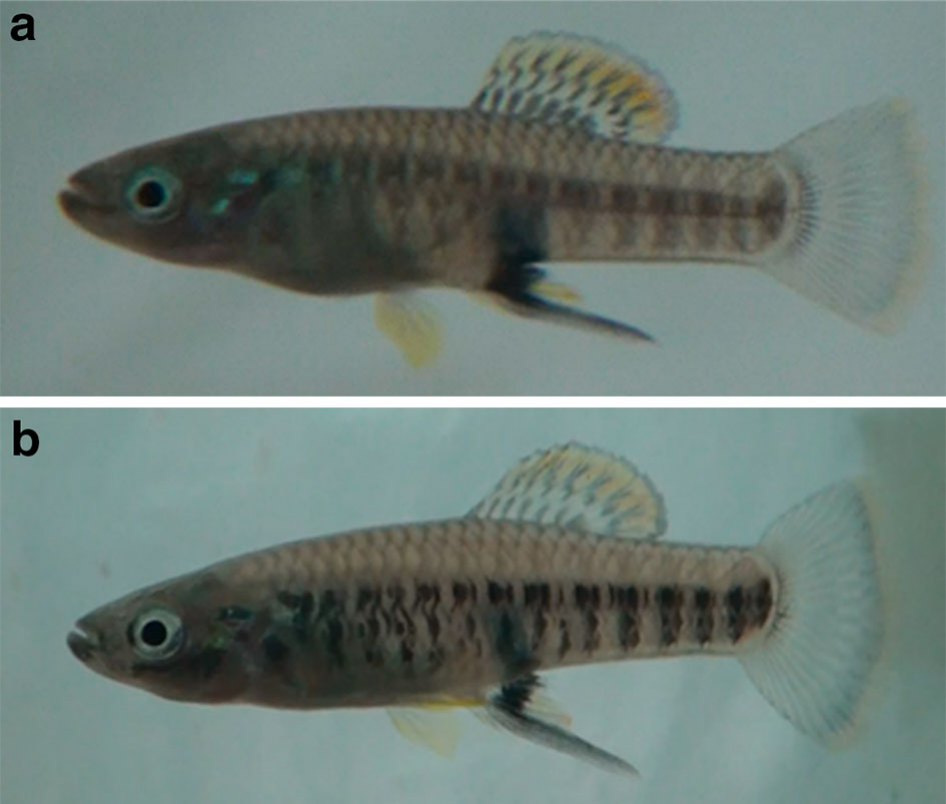
Change in body coloration in males. **a** Normal coloration of a male compared to **b** dark coloration in the same individual

Colour changes occurred in a male’s body during mating behavior, beginning during the approximation phase; however, these changes did not occur when only aggressive behaviours were displayed. The darkness on the lower half of the bars at the sides of the body intensified (henceforth ‘dark coloration’; Fig. 6). During this phase, males sometimes performed touch, touch attempt or lateral display, but more frequently males performed T-LD. Lateral display alone was an uncommon behaviour performed only 11 times by six (30%) of the males and was performed usually previous to the most common T-LD, which was performed 68 times by 16 males (80%). Lateral display as well as T-LD sometimes finished when the male swam quickly towards the ventral side of the female and touched the female (similar to approximation). This movement of the male to the ventral side of the female was performed in 27% and 76% of the lateral display and T-LD, respectively; and in 36% (lateral display) and 94% (T-LD) of the times males performed these behaviours they displayed dark coloration. During lateral display males had either the dorsal fin folded or extended, but the dorsal fin was always folded in T-LD. Gonopodial swings occurred in many different contexts, even when the male–female pair did not have visual contact. Copulation and copulation attempt were performed by only four males, each behaviour once during the first minute of the corresponding trail.

**Fig. 7a, b:**
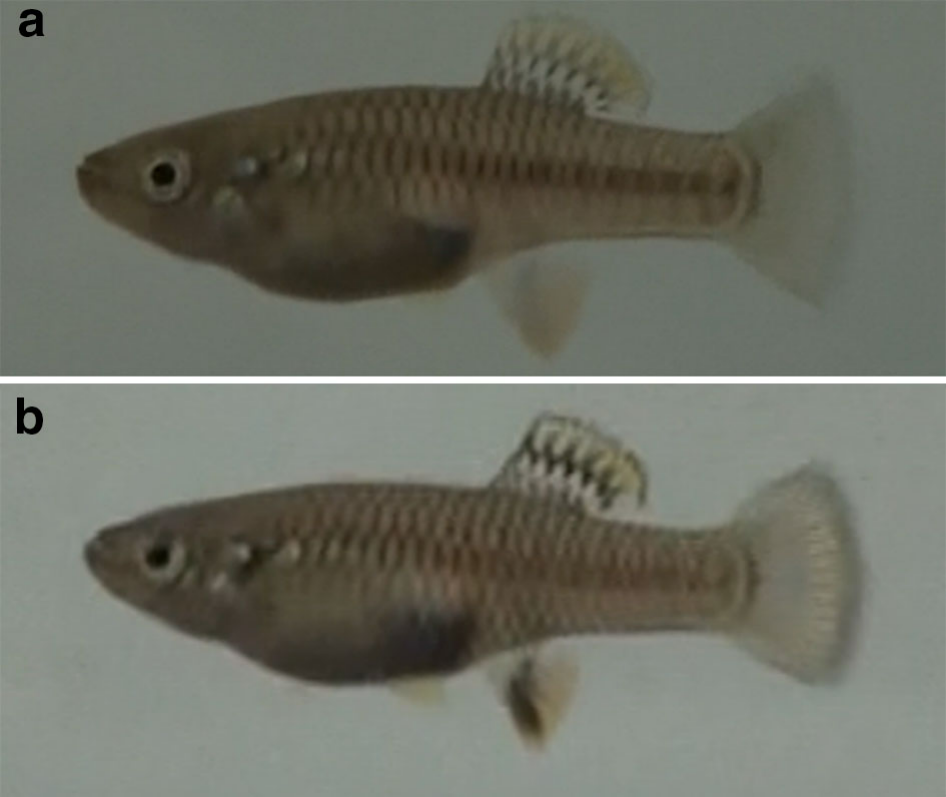
Change in anal fin coloration in females. **a** Normal coloration of a female’s anal fin compared to **b** a darker anal blotch

In females, the black blotch on the anal fin changed in shape and intensity in 14 trials (70% of trials; Fig. 7). These changes always occurred at the beginning of a trial, following the initial interactions with the male. The anal blotch darkened and expanded, covering the first anal rays (which were previously not covered by the dark spot) during the first minute of the interaction. Agonistic movements were more common in females than in males (Table 1) and were performed during aggressive interactions and the approximation phase. We did not observe any evident receptive display in females.

### Relations between female reproductive condition and behaviours

**Fig. 8a, b:**
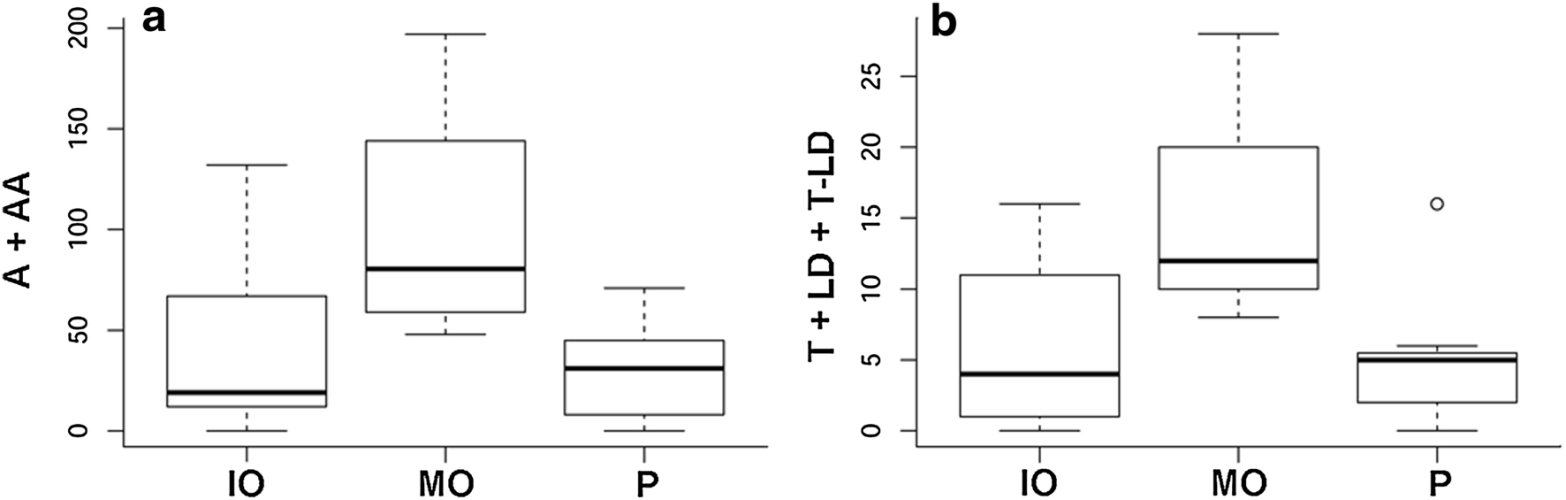
Box plot of a number of behaviours performed during each 20-min trial according to the reproductive condition of females [immature ovum (*IO*), mature ovum (*MO*), pregnant (*P*). **a** Approximation + approximation attempt (*A* + *AA*) of males, **b** touch + touch attempt + lateral display + touch-lateral display (*T* + *LD* + *T-LD*) of males

Males performed more approximation + approximation attempt (Fig. 8a) and touch + touch attempt + lateral display + T–LD (Fig. 8b; Tables 1, 2) to females with mature ovum than to females with other reproductive conditions (*t* = 2.41, *df* = 14, *p* = 0.0305 and *t* = 2.47, *df* = 14, *p* = 0.0269 respectively, Tables 1, 2). Males always performed mating behaviours to females with mature ovum, but some males did not perform mating behaviours to females with immature ovum and pregnant. Males did not perform Agonistic movements to females with mature ovum (Tables 1, 2). The length of males and females and their interaction (male length × female length) had no effect on males’ behaviours (Table 2).

**Table 2:** Effect of female reproductive condition (immature ovum, mature ovum and pregnant) on A + AA and T + LD + T-LD behaviours of males

Comparison	Value	SE	*t*_(14)_	*p*
A + AA				
Mature vs. immature	1.55	0.99	1.56	0.1419
Mature vs. pregnant	2.01	0.83	2.41	0.0305
Immature vs. pregnant	0.46	0.66	0.70	0.4936
Male length	5.57	4.25	1.31	0.2114
Female length	5.49	3.55	1.54	0.1445
Male × female length	−2.11	1.38	−1.52	0.1497
T + LD + T-LD				
Mature vs. immature	1.36	0.90	1.51	0.1533
Mature vs. pregnant	1.90	0.77	2.47	0.0269
Immature vs. pregnant	0.54	0.60	0.90	0.3843
Male length	5.08	3.90	1.30	0.2142
Female length	5.93	3.25	1.83	0.0893
Male × female length	−2.06	1.27	−1.62	0.1261

All comparison had 14* df*. For abbreviations, see Table 1

## Discussion

### Behavioural descriptions

*B. olomina* showed a wide range of aggressive and mating behaviours; some of these (coordinate, touch and lateral display) have not previously been described for this species (note ‘*B. olomina*’ included in *Brachyrhaphis rhabdophora* in Mojica 1996), or for other poeciliids (Table 3). Sidle spread and tail beating were considered by Mojica (1996) to be behaviours performed by males and females during aggressive encounters. Liley (1966) reported the behavioural sparring for four *Poecilia* species as an agonistic behaviour by males and females consisting of the two individuals having their longitudinal axes parallel and proceeding slowly forward with the body straight or slightly arched and fins fully spread while quivering. Males seldom performed sparring directed toward females (Liley 1966). Spread and sidle spread show some features similar to sparring: fins fully spread, body is slightly arched (weak sigmoid); they also tend to occur in male–male and female–female interactions (Garita-Alvarado, unpublished data). Contrary to sparring, males often perform sidle spread to females and usually perform it before the female. Sidle spread is a very conspicuous behaviour in which fins, body and gonopodium are displayed to the receiver fish, and sometimes there are no aggressive movements of the female between sidle spreads. This suggests a possible role of sidle spread in courtship or at least in showing body features prior to mating behaviours since fins and gonopodium are known to be under sexual selection in poeciliids (MacLaren and Rowland 2006; Langerhans 2011), and males performed more sidle spread than females. Alternatively, males might simply be more aggressive than females. Future tests on aggressive behaviour in trials of the same sex will give more insight on this. Additionally, Clark et al. (1954) described the S-curving behaviour performed by males and females of two *Xiphophorus* species, which also consists of arching the body with the dorsal and caudal fins completely extended, as a behaviour that correlates with establishing hierarchical status (Braddock 1945; Clark et al. 1954). The resemblance of this to sidle spread suggests a possible role of sidle spread in dominance-submission behaviour in *B. olomina*, but this needs to be tested, particularly considering that Mojica (1996) mentioned dominance-submission relations in males of *B. rhabdophora*.

**Table 3:** Comparison of behaviours described in this study for *Brachyrhaphis olomina* and those previously described for ‘*B. olomina*’ (=*Brachyrhaphis rhabdophora* in Mojica 1996) and for other poeciliids

Category of behaviour	This study	Mojica (1996)	Mojica (1996)	Clark et al. (1954)	Liley (1966)	Liley (1966)	Karplus and Algom (1996)
	*Brachyrhaphis olomina*	*Brachyrhaphis olomina* (as *Brachyrhaphis rhabdophora*)	*Brachyrhaphis roseni*	*Xiphophorus helleri, Xiphophorus maculatus*	*Poecilia picta*	*Poecilia reticulata, Poecilia parae, Poecilia vivipara*	*Gambusia holbrooki*
Aggressive behaviour	SS	SS	SS	S-curving^a^	Sparring^a^	Sparring^a^	
	Tail beating	Tail beating	Tail beating	Tail slapping	Tail beating	Tail beating	Tail beating
	Coordinate	–	–	–	–	–	–
	Agonistic movement	Chase, charge, bite	Chase, charge, bite	Nip	Attack	Attack	Nip, chase
Mating behaviour	A	Dance^a^	–	–	Dance^a^	–	–
	T	Vent-nudge^a^	Vent-nudge^a^	–	–	–	–
	LD	–	Flank^a^	–	–	–	–
	T-LD	–	–	–	–	–	–
	Gonopodial swing	Gonopodial swing	Gonopodial swing	Gonopodial swing	Gonopodial swing	Gonopodial swing	–
	Copulation	Thrust	Thrust	Thrust, copulation	Thrust, copulation	Thrust, copulation	–

For abbreviations, see Table 1

^a^Behaviours similar to those described in this study; see “Discussion” for explanation

Tail beating has been reported in the poeciliid genera *Xiphophorus* [as ‘tail slapping’ in Clark et al. (1954)], *Poecilia* (Liley 1966), *Brachyrhaphis* (Mojica 1996) and *Gambusia* (Karplus and Algom 1996) (Table 3). In *Brachyrhaphis*, Mojica (1996) only mentioned tail beating but did not discuss its possible function. Clark et al. (1954) considered tail slapping to be an avoidance response of females with a low level of sexual receptivity, and Liley (1966) also considered tail beating as an agonistic behaviour of both males and females. Similar to sidle spread, tail beating is an aggressive behaviour but could also play a role in showing body features prior to mating behaviours for males since they performed more tail beating than females in general (Table 1). Also, tail beating is a very conspicuous behaviour in which fins and body are displayed to the receiver, but this also needs to be tested in trials with the same sex. The coordinate behaviour was not reported in Mojica (1996) and it has not previously been described for any poeciliid. Coordinate could resemble a chase in response to sidle spread; however, in a chase, the fins are not extended (dorsal, caudal and gonopodium-anal fins are fully extended in the coordinate behaviour) and the behaviour of the fish is not coordinated. Coordinate could also resemble sparring (Liley 1966), in which both fish move slowly with fins fully spread and the body quivering. Coordinate differs to sparring because in coordinate the movement is very fast and the body does not quiver.

The behaviour dance described by Liley (1966) for *P. picta* and by Mojica (1996) for *B. olomina* (as *B. rhabdophora*) (Table 3) consists of ‘circling and figure-of-eight movements to and fro beneath the female’s head and belly’. We did not observe this behaviour in *B. olomina*, though it has some similarities with the approximation and approximation attempt, although it also differs from them: (1) dance includes circling and figure-of-eight movements, but during approximation the male swims below the female in very variable forms (but not specifically a figure-of-eight); and (2) the male sometimes stops between each approximation or approximation attempt while, according to Liley (1966), in dance the male performs the figure-of-eight in succession. Mojica (1996) described a behaviour that consisted of: ‘the male is watching the female from above and then quickly swims down to the female and performs a vent-nudge’. This behaviour is similar to the touch behaviour described here, but the site of contact differs since the male touches the female’s belly and the ventral side of the head, not necessarily the vent.

Touch and approximation are very quick movements and could be confused; however, there are differences between them:

1. In touch, the male swims to a position parallel to the longitudinal axis of the female so that both face in the same direction, while in approximation the male makes contact with the female at many different angles along the longitudinal axis of the female.
2. In touch, the male stays a short time in front of the female after touching it, while in approximation the male continues swimming after contact with the female.
3. In touch, the male longitudinal axis is inclined with the head upward at an angle of about 45° while touching the female, but in approximation the male’s longitudinal axis is not inclined.
4. In approximation, after contacting the female, the male faces the female again and usually performs approximation again (often several times), while touch is only performed once at a time.

The lateral display described here is similar in some respects to the flank behaviour described by Mojica (1996) for *B. roseni*: in both behaviours the male shows one side and then the other to the female. They differ, however, because in flank the male does not roll on its longitudinal axis, the dorsal fin is extended (the dorsal fin is usually folded in lateral display) and the male holds its position in the water column while displaying, but in lateral display the male moves towards the female when showing its side. As mentioned above, in *B. olomina*, touch and lateral display are often performed in succession and together represent a later stage of courtship (suggested by the differences with lateral display without the previous touch), which probably corresponds with a more excited condition of the male. For example, *P. reticulata* males change their behaviour during a more excited condition, spreading fully the caudal and dorsal fins during an ‘intense period of courtship’, rather than folding the fins, which is shown by males during less intense courtship displays (Liley 1966). There is no description of a behaviour similar to T–LD for any other poeciliid species.

Gonopodial swing is a behaviour shared by all poeciliid species studied (Rosen and Gordon 1953). There are two different ways to complete the forward movement of the gonopodium: using either the pectoral or the pelvic fin (Rosen and Tucker 1961; Peden 1972). *B. olomina* uses the pelvic fin to complete the gonopodial swing movement, as in *Xiphophorus* (Rosen and Gordon 1953) and *Poecilia* (Garita-Alvarado, personal observation). Also, the copulation mechanism is similar to that reported for other poeciliid species (Rosen and Gordon 1953).

### Mating behavioural sequence, frequency and colour changes

Mojica (1996) did not describe a sequence of behaviours for *B. olomina*, but found, as we did, that males performed sidle spread more often than copulation attempt. Mojica (1996) reported sidle spread as an aggressive behaviour and also reported that vertical bars appeared during aggressive encounters in males and females. However, we did not see any colour changes in male or female during sidle spread. Mojica (1996) described a colour change during an observation just before dance, which was similar to colour change displayed during the approximation phase described here for *B. olomina*. However, Mojica (1996) was not clear whether the entire vertical bars darkened, or only their lower halves, as we observed (Fig. 6).

Baerends et al. (1955) described the colour change of body markings during mating and agonistic behaviour for *P. reticulata*, as did Liley (1966) for three other *Poecilia* species. *P. parae* shows intense body markings during courtship, *P. picta* shows body markings only during aggressive encounters and *P. vivipara* does not change coloration (Liley 1966). In *P. reticulata*, body markings intensify during both courtship and agonistic behaviour between males, but the patterns of markings are different during courtship and aggression and also at different stages of male courtship (Baerends et al. 1955). Similar to *P. parae* and *P. reticulata*, colours darken in *B. olomina* during mating behaviour. Additionally, the change in the coloration of the anal fin in females of this species seems to be associated with aggressive behaviour. Mojica (1996) noted that individuals of *B. olomina* which were chased by other fish blanched the markings on their bodies and anal fin or gonopodium. We observed that anal fins became darker during female–female aggressive encounters (additional female–female observation; Fig. 4; http://www.momo-p.com/showdetail-e.php?movieid=momo170720bo02a).

### Female reproductive condition and behaviours

The behaviour that male *B. olomina* performed to females with mature ovum (more mating behaviour, no agonistic movements), but not to females with immature ovum and those which were pregnant suggests that males can detect a female’s reproductive condition. Theory predicts that the intensity of inter-male competition should correlate with female condition, and that the detection of female condition must be possible for this correlation to occur (Park and Propper 2002). Despite we did not test for inter-male competition in this study, there is some evidence of male–male aggression and dominance-submission interaction in this species (Mojica 1996; Garita-Alvarado, unpublished data). However, the adaptive advantage of inter-male competition for females and the specific mechanisms of detection of the female reproductive condition by males of *B. olomina* and other *Brachyrhaphis* species are unknown and should be addressed in future studies. We did not find a specific receptive behaviour in females, but it is common for at least half of the poeciliid species that females do not cooperate during mating (Bisazza 1993). Alternatively, it is possible that females need a longer time for habituation to the test aquarium to perform receptive behaviours, but this needs to be tested in future studies.

Although the behaviour of most species in the most diverse genera of poeciliids is still unknown, the mating behaviours reported in this study and for other poeciliids allow for some general ideas and hypotesis regards the evolution of mating behaviours across phylogenetically closely related genera. Pollux et al. (2014) found that *Brachyrhaphis* is not a monophyletic genus, but instead consists of two monophyletic groups (among the six species studied). One group included the ‘deeper body species’ (*Brachyrhaphis terrabensis, Brachyrhaphis rhabdophora, Brachyrhaphis roseni, Brachyrhaphis holdridgei*) and the other group the ‘slender body species’ (*Brachyrhaphis parismina and Brachyrhaphis cascajalensis*). Each group is more closely related to *Phallichthys* and *Priapichthys*, respectively, both genera in which courtship is absent. Both groups (*Phallichthys* and the deeper body species) and (*Priapichthys* and the slender body species) are closely related to *Alfaro*, a genus that presents courtship behaviour (Garita-Alvarado, unpublished data). In a biogeographic study on *Brachyrhaphis*, Ingley et al. (2015) also found that the ‘slender species group’ is closely related to *Priapichthys,* and that both the slender and deeper groups are monophyletic. This suggests that courtship was probably lost in *Phallichthys* and *Priapichthys,* but further analyses on specific mating behaviour of other genera closely related to the group (e.g. *Xenophallus, Heterandria*, Pullox et al. 2014) are required to have a better understanding of the evolution of the mating behaviour in this family. The lack of information on mating behaviour of the other *Brachyrhaphis* species (i.e., *Brachyrhaphis terrabensis, Brachyrhaphis parismina*, *Brachyrhaphis holdridgei*, *Brachyrhaphis hessfeldi, Brachyrhaphis roswitae* and *Brachyrhaphis puntifer*) prevent us from hypothesizing on the evolution of behaviour in this genus.

In conclusion, the mating and aggressive behaviours of *B. olomina* are complex and the new discoveries reported here highlight the importance of more species-specific studies and detailed description of the behaviour of poeciliids. Among the studied species of the family, some behaviours (e.g. tail beating) are apparently widespread, while others (touch, lateral display and coordinate) seem to be unique to *B. olomina*.

## References

[CR1] Angulo A, Garita-Alvarado CA, Bussing WA, López MI (2013). Annotated checklist of the freshwater fishes of continental and insular Costa Rica: additions and nomenclatural revisions. Check List.

[CR2] Baerends GP, Brouwer R, Waterbolk HT (1955). Ethological studies on *Lebistes reticulatus* (Peters). 1. An analysis of the male courtship pattern. Behaviour.

[CR3] Bisazza A (1993). Male competition, female mate choice and sexual size dimorphism in poeciliid fishes. Mar Behav Physiol.

[CR4] Bisazza A, Grapputo A, Nigro L (1997). Evolution of reproductive strategies and male sexual ornaments in poeciliid fishes as inferred by mitochondrial 16 rRNA gene phylogeny. Ethol Ecol Evol.

[CR5] Braddock JC (1945). Some aspects of the dominance-subordination relationship in the fish *Platypoecilus maculatus*. Physiol Zool.

[CR6] Brett BLH, Grosse DJ (1982). A reproductive pheromone in the Mexican poeciliid fish *Poecilia chica*. Copeia.

[CR7] Bussing WA (1998). Freshwater fishes of Costa Rica.

[CR8] Clark E, Aronson LR, Gordon M (1954). Mating behavior patterns in two sympatric species of xiphophorin fishes: their inheritance and significance in sexual isolation. Bull Am Mus Nat Hist.

[CR9] Crow RT, Liley NR (1979). A sexual pheromone in the guppy, *Poecilia reticulata* (Peters). Can J Zool.

[CR10] Farr JA (1975). The role of predation in the evolution of social behavior of natural populations of the guppy, *Poecilia reticulata* (Pisces: Poeciliidae). Evol.

[CR11] Farr JA, Travis J (1986). Fertility advertisement by female sailfin mollies, *Poecilia latipinna* (Pisces: Poeciliidae). Copeia.

[CR12] Ghedotti MJ (2000). Phylogenetic analysis and taxonomy of the poecilioid fishes (Teleostei: Cyprinodontiformes). Zool J Linn Soc.

[CR13] Hassell E, Meyers PJ, Billman EJ, Rasmussen JE, Belk MC (2012). Ontogeny and sex alter the effect of predation on body shape in a live-bearing fish: sexual dimorphism, parallelism, and costs of reproduction. Ecol Evol.

[CR14] Haynes JL (1995). Standardized classification of poeciliid development for life-history studies. Copeia.

[CR15] Ingley SJ, Johnson JB (2016). Selection is stronger in early-versus-late stages of divergence in a Neotropical live-bearing fish. Biol Lett.

[CR16] Ingley SJ, Reina RG, Bermingham E, Johnson JB (2015). Phylogenetic analyses provide insights into the historical biogeography and evolution of *Brachyrhaphis* fishes. Mol Phyl Evol.

[CR17] Johnson JB (2002). Divergent life histories among populations of the fish *Brachyrhaphis rhabdophora*: detecting putative agents of selection by candidate model analysis. Oikos.

[CR18] Karplus I, Algom D (1996). Polymorphism and pair formation in the mosquito fish *Gambusia holbrooki* (Pisces: Poeciliidae). Environ Biol Fish.

[CR19] Kolluru GR, Bertram SM, Chin EH, Dunmeyer CV, Graves JS (2014). Mating behavior and its morphological correlates in two color morphs of *Girardinus metallicus* (Pisces: Poeciliidae), a species previously thought not to exhibit courtship display. Behav Process.

[CR20] Langerhans RB, Evans J, Pilastro A, Schlupp I (2011). Genital evolution. Ecology and evolution of poeciliid fishes.

[CR21] Langerhans RB, DeWitt TJ (2004). Shared and unique features of evolutionary diversification. Am Nat.

[CR22] Leary S, Underwood W, Anthony R, Cartner S, Corey D, Grandin T, Greenacre C, Gwaltney-Brant S, McCrackin MA, Meyer R, Miller D, Shearer J, Yanong R (2013). AVMA guidelines for the euthanasia of animals.

[CR23] Liley NR (1966). Ethological isolating mechanisms in four sympatric species of poeciliid fishes. Behaviour.

[CR24] Lucinda PHF, Reis RE (2005). Systematics of the subfamily Poeciliinae Bonaparte (Cyprinodontiformes: Poeciliidae), with an emphasis on the tribe Cnesterodontini Hubbs. Neotrop Ichthyol.

[CR25] MacLaren DR, Rowland WJ (2006). Female preference for male lateral projection area in the shortfin molly, *Poecilia mexicana*: evidence for a pre-existing bias in sexual selection. Ethol.

[CR26] Martin SB, Albert JS, Leperg PL, Uribe CM, Grier HJ (2010). The evolution of the poeciliid gonopodium: integrating morphological and behavioral traits. Viviparous fishes II.

[CR27] Mojica CL (1996). The behavior of the live-bearing fish genus *Brachyrhaphis* in an ecological and historical context.

[CR28] Park D, Propper CR (2002). Pheromones from female mosquito fish at different stages of reproduction differentially affect male sexual activity. Copeia.

[CR29] Parzefall J (1969). Zur vergleichenden Ethologie verschiedener *Mollienesia*-Arten einschließlich einer Höhlenform von *M. sphenops*. Behaviour.

[CR30] Parzefall J, Schröder JH (1973). Attraction and sexual cycle of poeciliids. Genetics and mutagenesis of fish.

[CR31] Peden AE (1972). The function of gonopodial parts and behavioural pattern during copulation by *Gambusia* (Poeciliidae). Can J Zool.

[CR32] Peden AE (1975). Differences in copulatory behavior as partial isolating mechanisms in the poeciliid fish *Gambusia*. Can J Zool.

[CR33] Plath M, Makowicz AM, Schlupp I, Tobler M (2007). Sexual harassment in live-bearing fishes (Poeciliidae): comparing courting and noncourting species. Behav Ecol.

[CR40] Pollux BJA, Meredith RW, Springer MS, Garland T, Reznick DN (2014). The evolution of the placenta drives a shift in sexual selection in livebearing fish. Nature.

[CR34] Reynolds JD, Gross MD, Coombs MJ (1993). Environmental conditions and male morphology determine alternative mating behaviour in Trinidadian guppies. Anim Behav.

[CR35] Rosen DE, Gordon M (1953). Functional anatomy and evolution of genitalia of poeciliid fishes. Zoologica.

[CR36] Rosen DE, Tucker A (1961). Evolution of secondary sexual characters and sexual behavior patterns in a family of viviparous fishes (Cyprinodontiformes: Poeciliidae). Copeia.

[CR37] Schlosberg H, Duncan MC, Daitch BH (1949). Mating behavior of two live-bearing fish, *Xiphophorus hellerii* and *Platypoecilus maculatus*. Physiol Zool.

[CR38] Trivers R, Campbell B (1972). Parental investment and sexual selection. Sexual selection and the descent of man.

[CR39] Wang S, Cummings M, Kirkpatrick M (2015). Coevolution of male courtship and sexual conflict characters in mosquitofish. Behav Ecol.

